# Performance Assessment of a Locally Semi-Automated NGS-Based Workflow for Homologous Recombination Deficiency Testing in High-Grade Serous Ovarian Carcinoma

**DOI:** 10.3390/biomedicines14061405

**Published:** 2026-06-22

**Authors:** Maria Colomar-Roig, Lara Navarro, Javier Megías, Martín Núñez-Abad, Esther Roselló-Sastre, Nuria Santonja-López, Teresa San-Miguel

**Affiliations:** 1 Department of Pathology, Consorcio Hospital General Universitario de Valencia (CHGUV), 46014 Valencia, Spain; 2 Department of Pathology, Faculty of Medicine, Universitat de València, INCLIVA Biomedical Research Institute, 46010 Valencia, Spain; 3 Department of Medical Oncology, Consorcio Hospital General Universitario de Valencia (CHGUV), 46014 Valencia, Spain

**Keywords:** homologous recombination deficiency, *BRCA1*, *BRCA2*, ovarian cancer, NGS, SeqOne, SOPHiA genetics, Myriad MyChoice, HRD

## Abstract

**Background/Objectives**: Homologous recombination deficiency (HRD) is a predictive biomarker in high-grade serous ovarian carcinoma for platinum-based chemotherapy and PARP inhibitors. The implementation of HRD testing in routine diagnostics has generated multiple commercial assays that differ in genomic targets, bioinformatic analysis, and HRD scoring strategies. We aimed to assess the analytical performance and feasibility of a locally semi-automated workflow based on the Agilent SureSelect CD HRR17 panel with SeqOne/SomaHRD analysis, and to compare it with established commercial HRD assays currently used in routine clinical practice: Myriad MyChoice CDx and SOPHiA DDM Dx HRD Solution. **Methods**: Thirty high-grade serous ovarian carcinoma cases diagnosed between 2019 and 2023 were retrospectively analyzed. HRD status was assessed with the Agilent-SeqOne workflow and compared with Myriad (*n* = 12) and SOPHiA (*n* = 18). Concordance and correlation between genomic instability metrics were evaluated. **Results:** The Agilent/SeqOne workflow showed high concordance with both comparison workflows. Genomic instability metrics strongly correlated across assays (R^2^ up to 0.96). A lower proportion of inconclusive classifications was observed with the Agilent/SeqOne workflow. Discordances were mainly observed in borderline cases near classification thresholds. Variant detection was highly concordant within shared genomic regions. **Conclusions:** The locally semi-automated HRD workflow demonstrated high analytical concordance with established commercial assays in evaluable cases. Operational advantages related to workflow flexibility and local reanalysis support its potential implementation in routine molecular diagnostics.

## 1. Introduction

Ovarian cancer represents one of the most significant challenges in the field of gynecologic oncology due to its insidious and often asymptomatic nature in early stages, leading to late diagnosis and, consequently, relatively low survival [1,2]. Compared to other cancers, ovarian cancer is relatively uncommon in incidence but disproportionately lethal. It typically manifests between the ages of 55 and 64 and represents 3.0% of all new cancer diagnoses among women in Spain according to the Spanish Society of Medical Oncology (SEOM). The 5-year survival rate for ovarian cancer stands at 50.8% [3]. Translated to the Spanish population, this corresponds to approximately 3.700 new cases per year, with nearly half of these women expected to die within five years of diagnosis, at an age range that does not represent extreme aging. As a result, it is one of the leading causes of cancer death in women—comparable to lung, breast and colon cancer [4,5].

High-grade serous ovarian cancer (HGSOC) is the most prevalent subtype of ovarian cancer [6]. HGSOC is characterized by genetic instability and elevated mitotic activity. More than 96% of ovarian cancer cases exhibit mutations in the *TP53* gene. Additionally, a subset of HGSOC harbors alterations in *BRCA1* and *BRCA2*, which have distinct prognostic and therapeutic implications. Specifically, germline and somatic mutations in *BRCA1/2* are identified in 17–25% of patients, with somatic mutations accounting for 18–30% of all *BRCA1/2* mutations [7,8,9].

Moreover, approximately 50% of HGSOC cases exhibit homologous recombination deficiency (HRD) [10]. HRD is emerging as a significant factor in the biology of various cancers, most prominently ovarian, breast, pancreatic, and prostate cancer malignancies [11,12]. It represents a complex genomic signature that arises when cells fail to repair double-stranded DNA breaks through the homologous recombination repair (HRR) pathway [12]. The HRR pathway relies on multiple functionally interconnected genes, including *BRCA1* and *BRCA2* [11,13]. When the HRR pathway is compromised, double-strand breaks (DSBs) remain unrepaired or are incorrectly repaired via the error-prone non-homologous end-joining (NHEJ) pathway. This situation can lead to genomic instability, characterized by insertions, deletions, copy number variations, and structural chromosomal rearrangements, ultimately contributing to malignant transformation [14,15]. In HGSOC, the development of HRD is often attributed to loss-of-function mutations and epigenetic modifications in key genes involved in the HRR pathway. Beyond *BRCA1/2*, several other HRR-associated genes may contribute to the HRD phenotype: *ATM*, *BARD1*, *BRIP1*, *CHK1/2*, *CDK12*, *H2AX*, *MRE11*, *NBN*, *PALB2*, *RAD51B/C/D*, *RPA*, and the Fanconi Anemia Complementation Group genes, among others [16,17].

The clinical relevance of HRD testing is directly linked to the therapeutic success of PARP inhibitors. PARP inhibitors have shown particular efficacy in ovarian cancer patients harboring *BRCA1/2* mutations or those with defects in DNA repair pathways. They are also utilized in cases of disease relapse. Their mechanism of action involves the inhibition of alternative repair pathways apart from HRR, thereby inducing cell death in cells deficient in this pathway through synthetic lethality [18]. Clinical trials such as PRIMA, PAOLA1, and VELIA have demonstrated the clear benefits of PARP inhibitors Olaparib, Niraparib, and Veliparib in improving overall survival in ovarian cancer patients with homologous repair pathway deficiencies [9,19,20,21,22,23,24,25,26]. Consequently, the American Society of Clinical Oncology (ASCO) currently recommends offering germline and somatic genetic testing for *BRCA1/2* mutations to women diagnosed with epithelial ovarian cancer to guide appropriate treatment decisions [27]. Thus, HRD assays have recently been approved to stratify patients with wild-type *BRCA* and predict responses to platinum-based chemotherapy and synthetic lethality agents like PARP inhibitors [15].

As a result of this rapid clinical translation, HRD testing has moved from a research setting into routine molecular diagnostics. In controlled research and validation settings, HRD testing methods have demonstrated specific and reproducible performance characteristics under highly standardized conditions [28]. Commercial HRD assays differ substantially in target design, genomic instability metrics, degree of workflow automation, and regulatory status. CE-IVD-marked workflows such as Myriad MyChoice CDx and SOPHiA DDM™ Dx HRD Solution are increasingly incorporated into routine molecular diagnostics [29,30]. When implemented in routine clinical practice, assay performance and final HRD classification may differ from those observed in controlled environments [31]. However, other interesting solutions still remain as being for research-use-only (RUO). Locally deployable automated workflows based on sequencing panels and integrated bioinformatic analysis powered by artificial intelligence are emerging as potential alternatives for decentralized HRD testing.

We hypothesized that a locally semi-automated workflow based on the Agilent SureSelect CD HRR17 panel integrated with CE-IVD-marked SeqOne/SomaHRD analysis could achieve analytical concordance with established HRD testing workflows while improving workflow efficiency and reducing inconclusive results. This study aimed to assess the analytical performance and clinical feasibility of this automated workflow implemented under ISO 15189:2023 recommendations [32] by comparing HRD classification, genomic instability metrics, variant allele frequencies (VAFs), and somatic variant detection against two established reference assays, Myriad MyChoice CDx and SOPHiA DDM™ Dx HRD Solution.

## 2. Materials and Methods

### 2.1. Study Design and Cohort

We conducted a retrospective analytical study including 30 patients diagnosed with HGSOC at Consorcio Hospital General Universitario de Valencia (Valencia, Spain) between 2019 and 2023. The diagnosis of HGSOC was established according to routine histopathological evaluation by experienced pathologists, based on morphological criteria including marked nuclear atypia, elevated mitotic activity, and characteristic architectural features. Immunohistochemistry assessment was performed when required to support the diagnosis and exclude alternative histological subtypes. Samples were selected from a larger institutional cohort. Comparator HRD results corresponded to the assay that had been clinically performed at the time of routine patient management; therefore, not all cases had been analyzed using all three workflows. Inclusion criteria comprised confirmed HGSOC diagnosis, availability of sufficient formalin-fixed paraffin-embedded (FFPE) tumor material, tumor cellularity ≥ 20% and DNA yield adequate for NGS library preparation. Samples not meeting these criteria were excluded prior to molecular analysis. The study was conducted in accordance with the Declaration of Helsinki and approved by the Institutional Ethics Committee (CEIm) of CHGUV (protocol code 42/2024) and the CHGUV Biobank. Written informed consent for research use of samples and clinical data was obtained from all patients included in the study.

### 2.2. DNA Extraction

DNA was extracted from FFPE tissue sections of 10 µm thickness of representative tumor areas identified by a skilled pathologist, ensuring enough ≥20% tumor appropriated cellularity. Macrodissection was performed when necessary to enrich tumor content. Deparaffinization and tissue lysis were carried out using QIAGEN Deparaffinization Solution (QIAGEN GmbH, Hilden, Germany) and Sigma-Aldrich Proteinase K (Sigma-Aldrich, St. Louis, MO, USA). DNA extraction was performed using the QIAsymphony DSP DNA Mini Kit on the QIAsymphony SP Instrument (QIAGEN GmbH, Hilden, Germany). DNA concentration and purity were assessed using the Qubit DNA HS Assay Kit on a Qubit Fluorometer (Thermo Fisher Scientific, Waltham, MA, USA). Quality metrics and DNA integrity were evaluated following manufacturer’s guidelines to ensure suitability for NGS library preparation using the TapeStation system (Agilent Technologies, Santa Clara, CA, USA).

### 2.3. HRD Assessment Platforms and Workflows

**Figure 1:**
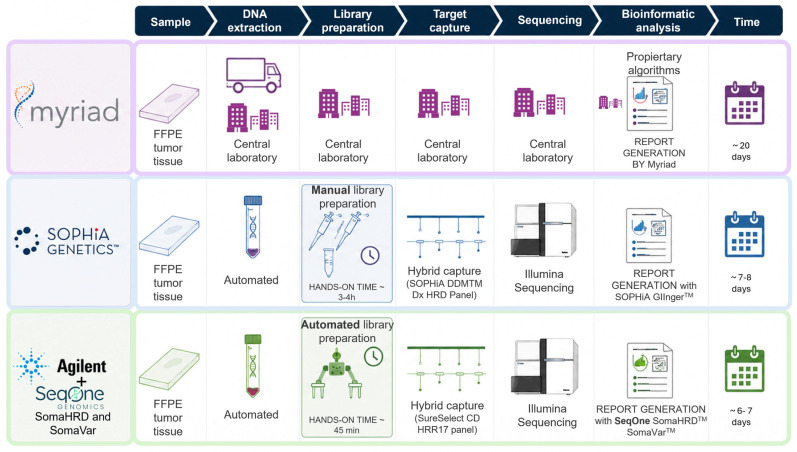
Overview of HRD assessment across the study cohort. HRD status was evaluated in 30 cases using the Agilent SureSelect CD HRR17 panel. Comparative HRD results obtained with CE-IVD reference assays were available for a subset of cases, including Myriad MyChoice CDx (*n* = 12) and SOPHiA DDM^TM^ Dx HRD Solution (*n* = 18).

HRD status was defined through the integrated assessment of pathogenic *BRCA1/2* alterations together with genome-wide measures of genomic instability, incorporated into HRD testing workflows, to generate a composite score reflecting homologous recombination deficiency. HRD status was assessed using the Agilent SureSelect CD HRR17 panel (Agilent Technologies, Santa Clara, CA, USA) in all 30 cases, which were bioinformatically analyzed with SeqOne software v1.2.0 and v1.3.15. For comparative purposes, HRD results obtained using CE-IVD reference assays were available for 12 cases (Myriad MyChoice CDx) and 18 cases (SOPHiA DDM^TM^ Dx HRD Solution). The evaluated HRD workflows differed with respect to automation level, externalized versus local processing steps, manual intervention requirements, and estimated turnaround time (Figure 1).

#### 2.3.1. Agilent SureSelect CD HRR17 Panel

HRD analysis was performed using Agilent SureSelect CD HRR17 panel. Library preparation and hybrid captures were conducted using the Agilent Magnis NGS Prep System according to the manufacturer’s instructions. Sequencing was performed on a NextSeq 550 platform (Illumina, San Diego, CA, USA). Raw sequencing data were analyzed using the SeqOne bioinformatic platform (SeqOne Genomics, Montpellier, France). It includes the SomaVar variant calling module to interrogate coding regions of 17 HRR-related genes (*BRCA1*, *BRCA2*, *BRIP1*, *CHEK1*, *CHEK2*, *PALB2*, *RAD51C*, *RAD51D*, *ARID1A*, *ATM*, *BRAF*, *CDK12*, *FANCA*, *FANCL*, *NBN*, *PIK3CA* and *TP53*) and the SomaHRD module (CE-IVD, versions 1.2.0 and 1.3.15) to assess the HRD probability (pHRD). According to the software thresholds, samples with pHRD ≥ 0.5 were considered HRD, whereas samples with pHRD < 0.5 were considered proficient for homologous recombination (HRp). pHRD was considered non-informative when confidence criteria defined by the algorithm version were not fulfilled, including a robustness of genomic instability value <0.85 (SomaHRD v1.2.0) or a pHRD confidence interval crossing the 0.5 cutoff (SomaHRD v1.3.15). Final HRD classification integrated both pHRD estimation and *BRCA1/2* status. Accordingly, samples with pathogenic *BRCA1/2* variants could still be classified as HRD despite non-informative pHRD estimates. Cases were finally classified as inconclusive when pHRD estimation was non-informative and no pathogenic or likely pathogenic *BRCA1/2* variants were identified.

#### 2.3.2. Myriad MyChoice CDx Workflow

HRD status for 12 cases had been previously determined using the CE-IVD-marked Myriad MyChoice^®^ CDx assay (Myriad Genetics, Salt Lake City, UT, USA). Testing was performed in a centralized reference laboratory according to the manufacturer’s validated protocol. The assay determines *BRCA1/2* variant status and calculates a Genomic Instability Score (GIS) based on telomeric allelic imbalance, large-scale state transitions, and loss of heterozygosity. Tumors with GIS ≥ 42 were classified as HRD. Consistent with the centralized service model, only the final HRD classification and variant clinical report were provided by the manufacturer, precluding access to raw sequencing data or reanalysis through independent bioinformatic pipelines.

#### 2.3.3. SOPHiA DDM™ Dx HRD Solution Workflow

HRD status for 18 cases had been previously assessed by us using the CE-IVD-marked SOPHiA DDM^TM^ Dx HRD Solution (SOPHiA GENETICS, Saint-Sulpice, Switzerland). Library preparation and hybrid captures were conducted manually according to the manufacturer’s instructions. Sequencing was performed on a NextSeq 550 platform (Illumina, San Diego, CA, USA). Raw sequencing data were analyzed using SOPHiA DDM software (version 5.10; HRD_v1), with GRCh37/hg19 as a reference genome. The assay integrates targeted sequencing of 28 HRR-associated genes with low-pass whole-genome sequencing. HRD classification was generated using the GIInger^TM^ deep learning-based algorithm to calculate the Genomic Integrity Index (GII). Tumors with GII ≥ 0 were classified as HRD.

### 2.4. Variant Classification and Comparisons

Detected variants were annotated according to ACMG and ENIGMA guidelines criteria and cross-referenced with ClinVar and OncoKB. Variant allele frequency (VAF) was recorded to evaluate concordance across workflows. Comparative analysis was performed including only cases meeting workflow-specific quality thresholds and yielding a conclusive HRD classification.

### 2.5. Statistical Analysis

Positive Percent Agreement (PPA), Negative Percent Agreement (NPA), and Overall Percent Agreement (OPA) were calculated to evaluate the performance of the Agilent SureSelect CD HRR17 panel compared with that of the Myriad MyChoice CDx and SOPHiA DDM™ Dx HRD Solution. Cohen’s Kappa (k) statistics were also calculated to assess the level of agreement between the different solutions and interpreted according to Landis and Koch criteria [33]. Confidence intervals of 95% were calculated for agreement metrics.

## 3. Results

### 3.1. Cohort Overview

A total of 30 patients diagnosed with HGSOC at the Consorcio Hospital General Universitario de Valencia between 2019 and 2023 were included. Genomic DNA was extracted from FFPE tumor samples with concentrations ranging from 2.1 to 190 ng/μL (median 50 ng/μL), all exceeding the minimum input recommended for the different assays. All libraries were within the manufacturer-recommended fragment size range for FFPE-derived DNA (200–400 bp).

All 30 patients were analyzed at our institution using semi-automated workflow based on the Agilent SureSelect CD HRR17 panel, SeqOne bioinformatic analysis, and SomaHRD/SomaVar interpretation tools. For cross-workflow comparison, HRD results obtained using Myriad MyChoice CDx were available for 12 cases, whereas 18 cases had been previously analyzed using the SOPHiA DDM™ Dx HRD Solution. Samples with inconclusive HRD classification and no pathogenic variants were excluded from pairwise concordance analyses. An overview of HRD classification results across the three evaluated workflows is provided in Table 1.

### 3.2. HRD Classification by Workflow

#### 3.2.1. HRD Classification by Agilent SureSelect CD HRR17 Panel with SeqOne/SomaHRD Analysis

All 30 samples were processed and analyzed locally using the Agilent/SeqOne workflow. Based on the final interpretation using SomaHRD v1.3.15, 18 samples were classified as HRD, 9 as homologous recombination-proficient (HRp), and 3 as inconclusive (#6, #22, and #30), representing 10% of the cohort.

Among HRD-classified tumors, nine fulfilled both *BRCA1/2* pathogenic variant and pHRD positivity criteria, two were classified as HRD based exclusively on *BRCA1/2* status, and seven were classified based on pHRD positivity alone. pHRD estimation by SomaHRD was non-informative in three samples (#6, #11, and #30) due to low-confidence genomic instability estimates. In sample #11, the presence of a pathogenic *BRCA1* variant allowed final classification as HRD despite the non-informative pHRD result. Sample #22, initially classified as HRD in SomaHRD v1.2.0, shifted to inconclusive after implementation of v1.3.15 due to its borderline pHRD value, highlighting the uncertainty in borderline genomic-scar cases (Appendix A Table A1).

#### 3.2.2. HRD Classification by Myriad MyChoice CDx Assay

Twelve samples were analyzed. Nine were classified as HRD, one as HRp, and two as inconclusive (#1 and #18) representing 16% of the cohort. All HRD-classified tumors showed elevated GIS, ranging from 48 to 83. Six of the HRD tumors harbored pathogenic *BRCA1/2* alterations, four affecting *BRCA1* and two *BRCA2*, while the remaining three HRD cases were *BRCA1/2* wild-type. The single HRp sample and both inconclusive cases were *BRCA1/2* wild-type. As the assay was performed in a centralized laboratory, only final HRD classification and *BRCA* reports were available.

#### 3.2.3. HRD Classification by SOPHiA DDM™ Dx HRD Solution

Eighteen samples were analyzed. Eleven were classified as HRD, four as HRp, and three as inconclusive (#7, #8, and #27), representing again 16% of the cohort. HRD status was determined using a GII cutoff of 0. The three samples ultimately reported as inconclusive were classified as such for different reasons. Samples #7 and #8 showed near-diploid genomic profiles with minimal copy number deviation, resulting in non-informative GII estimates. Three additional samples also yielded non-informative GII result estimates because of similarly low genomic instability; however, pathogenic *BRCA1/2* variants were detected in these cases, leading to final HRD classification. In contrast, sample #27 could not be reliably evaluated because high background noise related to poor sample quality interfered with the analysis.

Overall, among the HRD tumors, seven harbored pathogenic *BRCA1/2* alterations, six in *BRCA1* and one in *BRCA2*, while the remaining four HRD cases were *BRCA1/2* wild-type. All HRp and inconclusive samples were *BRCA1/2* wild-type.

### 3.3. Pairwise HRD Concordance Across Workflows

The overall concordance for HRD classification was evaluated by comparing Agilent SureSelect CD HRR17/SeqOne workflow results against the combined reference set, comprising 18 samples analyzed with Sophia Genetics and 12 samples with Myriad MyChoice. Using SomaHRD v1.2.0, agreement was high with PPA: 94.74%, NPA: 100%, OPA: 95.65% (95% CI, 78.05–99.89%) and a Cohen’s kappa coefficient of 0.86. After re-analysis with SomaHRD v1.3.15, sample #22 was reclassified as inconclusive and excluded from the evaluable cohort, resulting in complete agreement across all evaluable samples. Differences between workflows were mainly related to the handling of borderline or low-signal cases, which contributed to inconclusive classifications.

**Figure 2:**
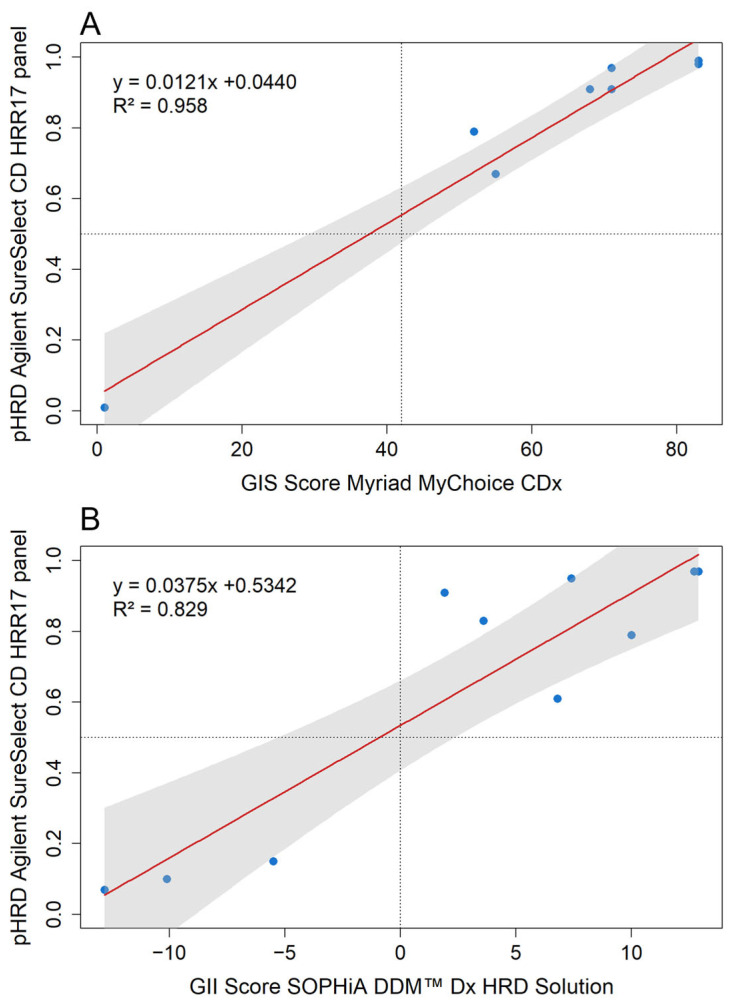
Correlation between continuous genomic instability metrics across workflows. (**A**) Linear regression analysis between the Genomic Instability Score (GIS) from Myriad MyChoice CDx and HRD probability (pHRD) generated by SomaHRD. (**B**) Linear regression analysis between the Genomic Integrity Index (GII) from SOPHiA and HRD probability (pHRD) generated by SomaHRD. Each dot represents an individual tumor sample; red lines indicate the fitted regression lines, and shaded areas represent the corresponding 95% confidence intervals. The continuous metrics showed strong proportional agreement across workflows despite differences in computational modeling.

Linear regression analyses demonstrated strong correlations between genomic instability metrics generated across workflows. Comparison between GIS values from Myriad MyChoice CDx and pHRD estimates generated by SeqOne/SomaHRD showed a strong relationship (R^2^ = 0.9582, Figure 2A). Similarly, GII values obtained with SOPHiA workflow strongly correlated with SeqOne/SomaHRD pHRD estimates (R^2^ = 0.82, Figure 2B).

#### 3.3.1. Comparison of HRD Between the Agilent/SeqOne Workflow and Myriad MyChoice CDx

Agreement between Agilent SureSelect CD HRR17 panel/SeqOne-SomaHRD workflow and Myriad MyChoice CDx assay was assessed in the subset of samples analyzed by both workflows (*n* = 12). Among these, seven tumors were concordantly classified as HRD and one as HRp, while four samples yielded inconclusive results in at least one workflow (Table 2). Samples with inconclusive classification in either workflow (#1, #6, #18, and #30) were excluded from the concordance analysis to ensure that agreement metrics were calculated only among cases with interpretable HRD results. After exclusion, eight samples remained evaluable for concordance analysis.

Within this subset, PPA, NPA, and OPA for HRD classification were 100%. The 95% CI for OPA was 63.06–100%. The Cohen’s kappa coefficient was also 1.00, indicating perfect agreement between both approaches.

*BRCA* variant detection showed near-complete concordance between workflows. Among the 12 samples analyzed with the Myriad MyChoice CDx assay, one sample (#24) was excluded due to insufficient coverage of the panel genes. Among the remaining 11 evaluable cases, a single discordant finding was observed (patient #4), in which Myriad detected a *BRCA1* exon 8–13 deletion. It was not identified by the Agilent/SeqOne workflow, probably reflecting the limited sensitivity of targeted NGS approaches for detecting large genomic rearrangements. Detailed *BRCA* variant information and cross-workflow comparison are provided in Appendix A Table A1.

#### 3.3.2. Comparison of HRD Between Agilent/SeqOne Workflow and SOPHiA DDM™ Dx HRD Solution

Agreement between Agilent SureSelect CD HRR17 panel/SeqOne-SomaHRD workflow and the SOPHiA DDM^TM^ Dx HRD Solution was evaluated in the subset of 18 samples analyzed by both workflows (Table 3).

**Figure 3:**
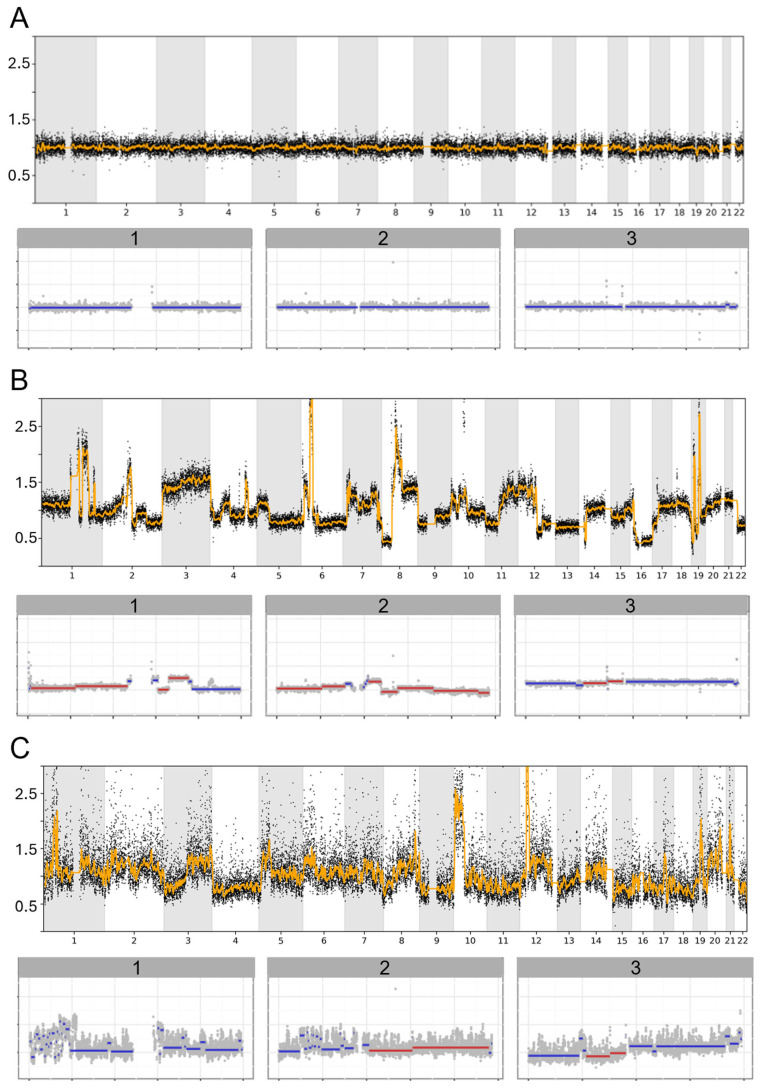
For each panel (**A**–**C**), the upper plot corresponds to SOPHiA GII analysis and the lower plot to SomaHRD pHRD analysis (red: segments displaying large-scale state transitions; blue: segments without large-scale state transitions). (**A**) Near-diploid genomic profile with minimal copy number deviation and limited genomic scarring. (**B**) Borderline genomic instability profile close to classification thresholds. (**C**) Low-quality sample with elevated background noise affecting confidence metrics.

Three samples (#7, #8, and #27) yielded inconclusive results with the SOPHiA workflow and were excluded from concordance analysis. Samples #7 and #8 showed near-diploid genomic profiles with minimal copy number deviation, resulting in non-informative GII estimates. In contrast, sample #27 presented high background noise that interfered with reliable genomic instability assessment. An additional borderline sample (#22) was initially classified as HRD by SomaHRD v1.2.0 and HRp by SOPHiA. However, after re-analysis using SomaHRD v1.3.15, this case was reclassified as inconclusive due to revised variability estimation across genomic windows and was therefore excluded from the final concordance analysis. Representative genomic profiles associated with inconclusive HRD classification are shown in Figure 3.

After exclusion of non-informative cases, 14 samples remained evaluable. Within this subset, concordance between workflows was complete, with PPA, NPA, OPA, and Cohen’s kappa values of 100%. The 95% CI for OPA was 76.84–100%.

Regarding *BRCA* status, all 18 samples analyzed with the SOPHiA workflow showed complete concordance with SomaVAR variant detection.

### 3.4. Peripheral Blood Analysis and Germline Verification

Peripheral blood analysis was performed to confirm the constitutional origin of *BRCA* variants identified in tumor sequencing. Germline *BRCA1/2* variants were confirmed in six patients, whereas two patients harbored somatic *BRCA* variants. One patient carried a *BRCA* variant preliminary classified as a variant of uncertain significance (VUS), and germline confirmation remained pending in four additional cases (Appendix A Table A1). Among all pathogenic variants detected, the majority were frameshift alterations, predominantly affecting *BRCA1* exon 10, which is the largest exon and accounts for approximately 61.3% of the coding sequence according to ClinGen ENIGMA *BRCA1* and *BRCA2* Expert Panel specifications [34].

The inclusion of germline testing serves as an internal validation step for somatic variant annotation within HRD analyses. Variants initially detected in tumor sequencing and subsequently confirmed in peripheral blood may be confidently classified as germline, supporting the accuracy of the variant calling and annotation workflow. This complementary analysis reduced the risk of misclassification and improved the interpretability of HRD characterization across the cohort. In addition, determining whether *BRCA1/2* alterations are present at the germline level is clinically relevant because it has implications not only for treatment selection and prognosis, but also for hereditary cancer risk assessment and genetic counseling of family members.

### 3.5. Somatic Variant Concordance and Integrated Mutational Landscape

To characterize the somatic mutational landscape associated with HRD, pathogenic and likely pathogenic variants were compared across two targeted NGS solutions: the SOPHiA DDM™ Dx HRD solution and the Agilent SureSelect CD HRR17 panel analyzed using SeqOne/SomaVar.

**Figure 4:**
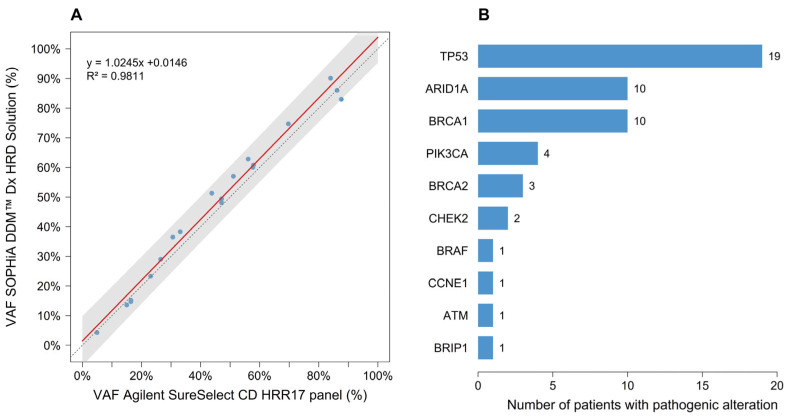
Somatic variant concordance and integrated mutational landscape across the cohort. (**A**) Linear regression analysis of variant allele frequencies (VAFs) obtained with the Agilent SureSelect CD HRR17/SeqOne-SomaVar workflow and the SOPHiA DDM™ Dx HRD workflow. Each point represents one shared variant. The red line indicates the fitted regression model, the shaded area the 95% prediction interval, and the gray dashed line the line of identity (y = x). (**B**) Distribution of pathogenic or likely pathogenic alterations across the cohort. Variants were aggregated across workflows and redundant gene-level calls within the same sample were excluded.

All pathogenic/likely pathogenic variants were concordant between workflows for genes represented in both panels. Discrepancies were exclusively due to panel design: *PTEN* was absent from the Agilent panel, whereas *ARID1A* was excluded from the SOPHiA DDM™ Dx HRD panel. No discordant variant calls were observed in overlapping genomic regions. This highlights the importance of panel composition in cross-workflows comparisons. Quantitative concordance of variant allele frequency (VAF) measurements was assessed using linear regression restricted to variants detected by both workflows. A strong correlation was observed (R^2^ = 0.98), indicating highly consistent VAF estimation across workflows (Figure 4A). A slight overestimation of allele frequencies was observed with the Agilent/SeqOne workflow compared with the SOPHiA workflow.

*TP53* was the most frequently altered gene, with 19 pathogenic alterations, consistent with its established role as the dominant genomic event in HGSOC [35]. *BRCA1* and *ARID1A* followed, with 10 alterations each. Less frequent alterations involved *PIK3CA*, *BRCA2*, and *CHEK2*, whereas *BRIP1*, *ATM*, and *BRAF* alterations were identified only in isolated cases (Figure 4B).

Of note, the single case harboring *CCNE1* amplification (patient #27) showed rapid clinical progression and early death after diagnosis. *CCNE1* amplification and *BRCA* alterations are typically considered mutually exclusive events in HGSOC and have been associated with poor prognosis and primary resistance to platinum-based chemotherapy [36].

## 4. Discussion

Evaluating the HRD status has become increasingly relevant for the clinical management of ovarian cancer patients. Up to 50% of HGSOCs show defects in the HRR pathway [10] and they can benefit from PARP inhibitor therapies [9,19,20,21,22,23,24,25,26]. Consequently, reliable and reproducible HRD assessment is increasingly required in routine molecular diagnostics.

In this study, we evaluated the analytical performance and feasibility of a local and semi-automated workflow based on the Agilent SureSelect CD HRR17 panel with SeqOne/SomaHRD analysis, and compared it with two established HRD workflows, Myriad MyChoice CDx and SOPHiA DDM™ Dx HRD Solution. Despite the limited cohort size, concordance between workflows was high among evaluable cases, with a slight reduction in inconclusive results observed.

Interestingly, most discordant results were observed in tumors with either minimal copy number alterations or borderline genomic instability profiles. Samples #7 and #8 showed near-diploid genomic patterns with minimal copy number deviation, resulting in non-informative GII estimation by SOPHiA. These findings are consistent with previously described limitations of genomic-scar-based approaches in low-signal tumors [37]. Borderline cases also highlighted the impact of algorithmic variability. Sample #22 showed different classifications depending on the software version and analytical workflow used. These observations suggest that HRD classification in borderline cases may be influenced by bioinformatic parameterization, confidence thresholds, and software updates, even when the underlying genomic data remain unchanged. An additional advantage of locally implemented workflows is the possibility of reanalysis after software updates or variant reinterpretation. In our series, reanalysis with SomaHRD v1.3.15 modified the classification of selected borderline cases and improved the interpretation of uncertainty estimates. Unlike externalized workflows such as Myriad, local control of sequencing and bioinformatic analysis facilitates the traceability, iterative review, and incorporation of updated variant interpretation.

Concordance for *BRCA1/2* variant detection was high across workflows. Only one discordant case was identified, corresponding to a *BRCA1* exon 8–13 deletion detected by Myriad but not by the Agilent/SeqOne workflow. This discrepancy most likely reflects the limited sensitivity of this targeted NGS approach for detecting large genomic rearrangements in FFPE samples [38,39]. Complementary techniques such as MLPA or ddPCR remain valuable for confirming these alterations in clinical practice [40,41]. It is worth noting that additional HRR-related genes implicated in HRD biology [16,17] showed comparable variant allele frequencies across workflows, supporting reproducibility at the somatic variant level.

From a practical perspective, the Agilent/SeqOne workflow showed several features of interest for routine implementation, including lower DNA input requirements, lower tumor cellularity thresholds, and reduced hands-on time compared with the SOPHiA workflow. Our observations are also consistent with the clinically validated SeqOne approach reported by Boidot et al. in the PAOLA-1 cohort [42]. While that study focused on clinical validation in a large trial population, our work addresses practical aspects of local workflow implementation in routine molecular diagnostics. This study has important limitations, particularly the reduced cohort size and the incomplete overlap between compared workflows. Not all samples were analyzed using all three HRD assays, since comparator results reflected the HRD test previously performed during routine clinical implementation rather than a prospectively harmonized testing design. Consequently, direct concordance between the two reference workflows could not be assessed, and inconclusive comparator cases were not systematically retested using an alternative HRD assay. Therefore, the results should therefore be interpreted within the context of a retrospective implementation study rather than a fully paired cross-platform validation. In addition, the high agreement metrics observed after exclusion of inconclusive cases should be interpreted cautiously given these limitations. However, the operational advantages observed for the Agilent/SeqOne workflow may facilitate its integration into routine molecular diagnostics. The present study was not designed to evaluate associations between HRD classification and clinical outcomes such as PARP inhibitor response, platinum sensitivity, progression-free survival, or overall survival. Orthogonal genomic or functional validation approaches, including MLPA, SNP arrays, whole-genome sequencing, or RAD51 foci assays, were also beyond the scope of the present study. Future studies in larger clinical cohorts will be necessary to explore the predictive clinical value of this workflow. Although further studies in larger cohorts will be necessary to better characterize concordance in borderline and low-signal cases, this type of locally implemented workflow may become relevant as HRD stratification expands beyond ovarian cancer to breast, pancreatic, and prostate carcinomas [43], where genomic scarring metrics are increasingly incorporated into therapeutic algorithms.

## 5. Conclusions

In summary, our results support the feasibility of implementing a locally semi-automated HRD workflow with high concordance relative to established commercial assays in evaluable cases. Beyond analytical performance, local implementation may offer practical advantages related to workflow flexibility, turnaround time, and reanalysis capability. Although larger validation studies are still required, this semi-automated approach may facilitate the incorporation of HRD testing into routine molecular diagnostics, offering a middle ground between fully externalized testing strategies and entirely manual internal workflows.

## Figures and Tables

**Table 1:** Overview of HRD classification results across the evaluated workflows.

		Workflow				Workflow	
ID	Agilent/SeqOne	Myriad	SOPHiA	ID	Agilent/SeqOne	Myriad	SOPHiA
1	HRp	IN	NA	16	HRD	NA	HRD
2	HRp	HRp	NA	17	HRp	NA	HRp
3	HRD	HRD	NA	18	HRp	IN	NA
4	HRD	HRD	NA	19	HRD	NA	HRD
5	HRD	HRD	NA	20	HRD	NA	HRD
6	IN	HRD	NA	21	HRD	HRD	NA
7	HRp	NA	IN	22	IN	NA	HRp
8	HRp	NA	IN	23	HRD	HRD	NA
9	HRD	NA	HRD	24	HRD	HRD	NA
10	HRD	NA	HRD	25	HRD	NA	HRD
11	HRD	NA	HRD	26	HRD	NA	HRD
12	HRp	NA	HRp	27	HRp	NA	IN
13	HRD	NA	HRD	28	HRp	NA	HRp
14	HRD	NA	HRD	29	HRD	HRD	NA
15	HRD	NA	HRD	30	IN	HRD	NA

Abbreviations: HRD, homologous recombination deficiency; HRp, homologous recombination proficiency; IN, inconclusive; NA, not analyzed. The Agilent/SeqOne workflow comprised sequencing with the Agilent SureSelect CD HRR17 panel followed by SeqOne SomaHRD analysis. Myriad workflow corresponded to Myriad MyChoice CDx testing. SOPHiA workflow corresponded to SOPHiA DDM™ Dx HRD Solution analysis.

**Table 2:** Pairwise HRD concordance between the locally semi-automated Agilent/SeqOne workflow and Myriad MyChoice CDx.

	Myriad MyChoice CDx		
Agilent/SeqOne Workflow	HRD	HRp	Inconclusive
HRD	7	0	0
HRp	0	1	2
Inconclusive	2	0	0

Abbreviations: HRD, homologous recombination deficiency; HRp, homologous recombination proficiency.

**Table 3:** HRD classification concordance between the Agilent/SeqOne workflow and the SOPHiA DDM™ Dx HRD Solution (*n* = 18).

	SOPHiA DDM™ Dx HRD Solution		
Agilent/SeqOne Workflow	HRD	HRp	Inconclusive
HRD	11	0	0
HRp	0	3	3
Inconclusive	0	1	0

Abbreviations: HRD, homologous recombination deficiency; HRp, homologous recombination proficiency.

## Data Availability

The raw data supporting the conclusions of this article will be made available by the authors on request.

## Abbreviations

The following abbreviations are used in this manuscript:

CHGUV	Consorcio Hospital General Universitario de Valencia
GIS	Genomic instability score
GII	Genomic integrity index
HGSOC	High-grade serous ovarian cancer
HRD	Homologous recombination deficiency
HRR	Homologous recombination repair
HRp	Homologous recombination proficiency
NGS	Next-generation sequencing
pHRD	Homologous recombination probability

## Appendix A

**Table A1:** Comparative performance of HRD genomic panels. The table summarizes classification results across the evaluated workflows.

ID	Age	Tumor %	Myriad MyChoice CDx ^1^ and SOPHiA DDM™ Dx HRD Solution ^2^		Agilent SureSelect CD HRR17 Panel/SeqOne Analysis	
			GIS/GII	*BRCA* Mutation (VAF)	pHRD	*BRCA* Mutation (VAF)
01 ^1^	52	90		WT	0.17	WT
02 ^1^	54	95	1	WT	0.01	WT
03 ^1^	74	80	83	WT	0.99	WT
04 ^1^	35	85	68	*BRCA1* del exons 8–13	0.91	WT
05 ^1^	39	40	52	*BRCA1* c.2017G>T p.(Glu673*)	0.79	*BRCA1* c.2017G>T p.(Glu673*) 63.1%
06 ^1^	83	40	52	WT	0.58 ^LC^	WT
07 ^2^	77	75		WT	0.02	WT
08 ^2^	49	90		WT	0.02	WT
09 ^2^	41	85	3.6	WT	0.83	WT
10 ^2^	48	85	1.9	*BRCA1* c.68_69del p.(Glu23Valfs*17)	0.51	*BRCA1* c.68_69del p.(Glu23Valfs*17) 73.4%
11 ^2^	64	40		*BRCA1* c.5095C>T p.(Arg1699Trp)	0.09 ^LC^	*BRCA1* c.5095C>T p.(Arg1699Trp) 47.2%
12 ^2^	46	75	−12.8	WT	0.07	WT
13 ^2^	64	95	7.4	*BRCA1* c.1421T>A p.(Leu474*)	0.95	*BRCA1* c.1421T>A p.(Leu474*) 85%
14 ^2^	74	90	12.7	WT	0.97	WT
15 ^2^	55	80	12.9	*BRCA1* c.4065_4068del p.(Asn1355Lysfs*10)	0.97	*BRCA1* c.4065_4068del p.(Asn1355Lysfs*10) 70.8%
16 ^2^	65	85	10	WT	0.79	WT
17 ^2^	69	85	−10.1	WT	0.10	WT
18 ^1^	50	20		WT	0.09	WT
19 ^2^	76	90	1.9	*BRCA2* c.7558C>T p.(Arg2520*) 60%	0.91	*BRCA2* c.7558C>T p.(Arg2520*) 57.7%
20 ^2^	47	70		*BRCA1* c.1961del p.(Lys654Serfs*47) 14.7%	0.02	*BRCA1* c.1961del p.(Lys654Serfs*47) 16.4%
21 ^1^	56	70	83	*BRCA2* c.5091del p.(Phe1698Leufs*8)	0.98	*BRCA2* c.5091del p.(Phe1698Leufs*8) 62%
22 ^2^	73	85	−2.3	WT	0.52 ^LC^	WT
23 ^1^	57	95	71	*BRCA1* c.5266dup p.(Gln1756Profs*74)	0.91	*BRCA1* c.5266dup p.(Gln1756Profs*74) 66.1%
24 ^1^	40	90	71	*BRCA1* c.68_69del p.(Glu23Valfs*17)	0.97	na
25 ^2^	72	85		*BRCA1* c.4035del p.(Glu1346Lysfs*20) 83%	0.65	*BRCA1* c.4035del p.(Glu1346Lysfs*20) 87.6%
26 ^2^	40	70	6.8	WT	0.61	WT
27 ^2^	65	75		WT	0.06	WT
28 ^2^	67	80	−5.5	WT	0.15	WT
29 ^1^	53	85	55	*BRCA2* c.7033C>T p.(Gln2345*)	0.67	*BRCA2* c.7033C>T p.(Gln2345*) 70.8%
30 ^1^	48	90	48	WT	0.45 ^LC^	WT

Abbreviations: GIS: genomic instability score by Myriad; GII: genomic integrity index by SOPHiA; LC: low confidence; pHRD: HRD probability by SeqOne SomaHRD; WT: Wild-type. na: Not available. ^1^: Myriad workflow corresponded to Myriad MyChoice CDx testing. ^2^: SOPHiA workflow corresponded to SOPHiA DDM™ Dx HRD Solution analysis.
